# The 100 most-cited manuscripts in epilepsy epigenetics: a bibliometric analysis

**DOI:** 10.1007/s00381-023-06032-w

**Published:** 2023-06-20

**Authors:** Lijuan Fan, Lu Liu, Xueyi Rao, Xiaoqian Wang, Huan Luo, Jing Gan

**Affiliations:** 1 Department of Pediatrics, West China Second University Hospital, Sichuan University, No. 20, Section 3, South Renmin Road, Chengdu, 610041, Sichuan Province China; 2 Key Laboratory of Obstetric & Gynecologic and Pediatric Diseases and Birth Defects of Ministry of Education, Sichuan University, Chengdu, Sichuan China; 3 Key Laboratory of Development and Maternal and Child Diseases of Sichuan Province, Chengdu, Sichuan China; 4 West China School of Public Health, Sichuan University, Chengdu, Sichuan Province, China

**Keywords:** Bibliometric analysis, Citations, Epigenetics, Epilepsy, microRNA

## Abstract

**Purpose:**

The top citation article reflects the developmental milestone of a given field. The purpose of this bibliometric analysis was to identify and assess the 100 most-cited (T100) articles on the epigenetics mechanism of epilepsy.

**Methods:**

The Web of Science Core Collection (WoSCC) database was used to investigate, and search terms related to epilepsy epigenetics were compiled. Results were ranked according to citation number. The publication year, citation density, authorship, journal, country, institution, manuscript type, theme, and clinical topics were further evaluated.

**Results:**

The Web of Science search returned a total of 1231 manuscripts. The number of citations for a manuscript ranges from 739 to 75. The greatest number of manuscripts in the top 100 was published in the *Human Molecular Genetics and Neurobiology of Disease* (*n* = 4). The journal with the highest 2021 impact factor was *Nature Medicine* (IF = 87.244). The most-cited paper by Aid et al. reported a new nomenclature for mouse and rat BDNF gene and its expression profiles. Most manuscripts were original articles (*n* = 69), of which 52 (75.4%) report findings of basic scientific work. The most prevalent theme was *microRNA* (*n* = 29), and the most popular clinical topic was *temporal lobe epilepsy* (*n *= 13).

**Conclusions:**

The research on the epigenetics mechanism of epilepsy was in its infancy but full of potential. The developmental history and current achievements of hot themes, including *microRNA*, *DNA methylation*, and *temporal lobe epilepsy*, were overviewed. This bibliometric analysis provides useful information and insight for researchers when launching new projects.

**Supplementary Information:**

The online version contains supplementary material available at 10.1007/s00381-023-06032-w.

## Introduction

Epilepsy is one of the most common nervous system diseases. More than 70 million people worldwide suffer from epilepsy and 2.4 million people are newly diagnosed with epilepsy every year [1]. The current use of anti-epileptic drugs can only reduce the occurrence of seizures, but they do not treat the underlying pathophysiology of epilepsy [2].

**Fig. 1:**
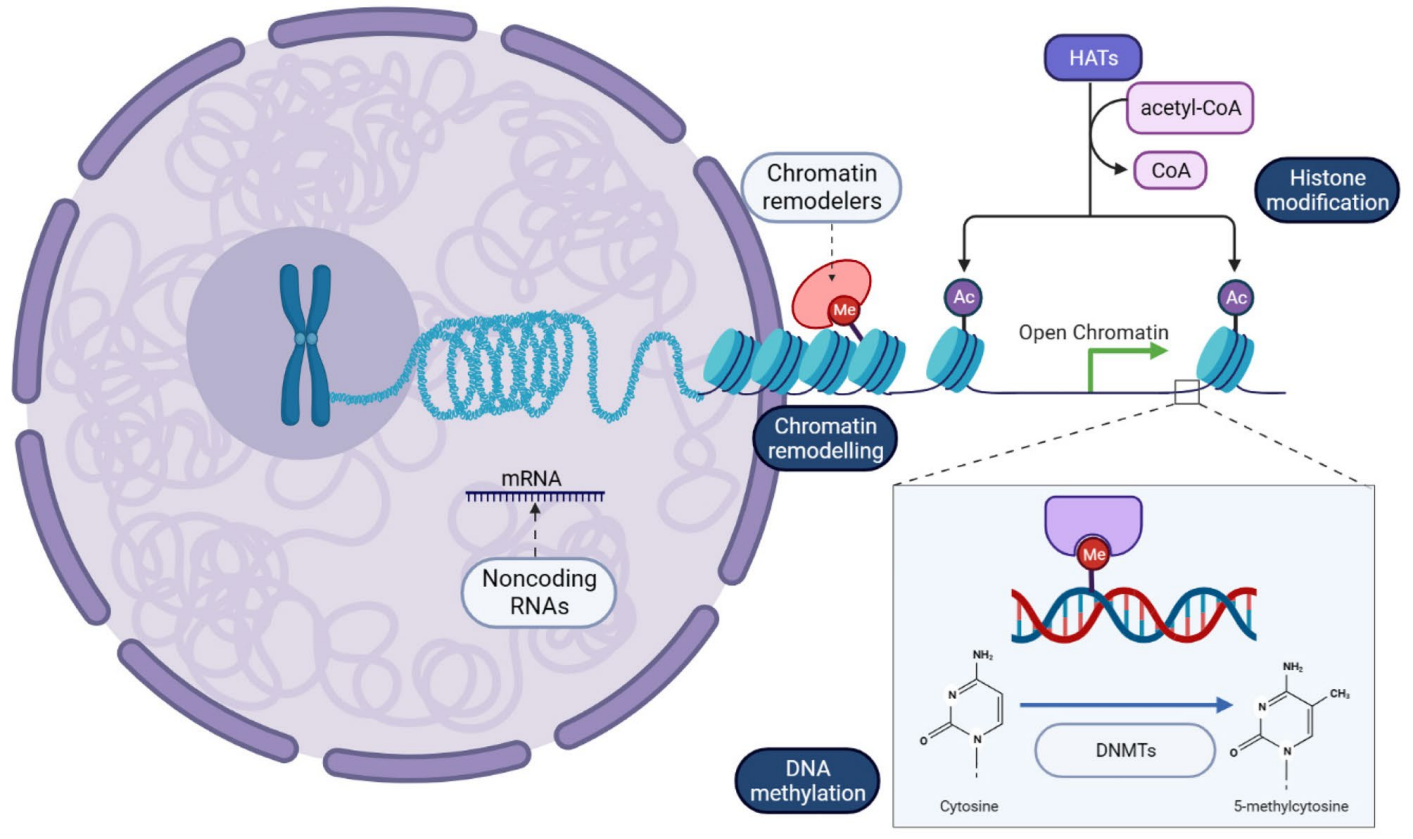
Brief scheme of epigenetic processes within the cell. Chromatin is comprised of nucleosomes (blue) that consist of DNA wrapped around a histone core. Chromatin remodeling is through the interaction between chromatin remodelers and modified histone proteins: Methylation (Me) of lysine residues on H3 leads to densely packed nucleosomes, thereby represses the gene expression, and acetylation (Ac) of histones executed by histone acetyltransferase (HATs) generally results in a more transcriptionally permissive structure. Methylation of DNA catalyzed by DNA methyltransferases (DNMTs) can promote or repress gene expression depending on methylation location and the transcription factors (TFs) recruited. In addition, noncoding RNAs in the cytoplasm can bind and degrade mRNA transcripts to regulate gene expression in post­transcriptional level

Epigenetics, introduced for the first time by Waddington in the early 1940s, has traditionally referred to a variety of mechanisms that allow heritable changes in gene expression without altering the genetic sequence [3]. However, with the fast expansion in this field, epigenetics has been redefined and accepted today as “The chemical modifications added to the entirety of one’s DNA (genome) as a way to regulate the activity (expression) of all the genes,” mainly composed of chromatin remodeling, histone modifications, DNA methylation, and non­coding RNAs (Fig. 1) [4]. The different categories of epigenetic mechanisms were not independent but worked in tandem. Chromatin remodelers can influence DNA methylation and histone modifications by condensing and loosening chromatin. Histone modifications can affect DNA methylation and vice versa. Furthermore, epigenetic proteins under the same epigenetic classification may affect each other [5].

Work that has the greatest impact on the scientific community is likely to be cited many times. Therefore, citation analysis is an important method to evaluate the impact of a research article. Over the years, many special fields have published bibliometric analyses on the most-cited papers in their speciality, which include abdominal surgery [6], urological surgery [7], bladder cancer [8], pediatric dentistry [9], and glioblastoma multiforme [10], but none has specifically focused on epigenetic mechanisms’ underlying epilepsy.

Understanding the contribution of epigenetic changes to epilepsy will hopefully provide us with better molecular tools for an improved diagnosis and therapy of these patients. Therefore, the purpose of this study was to analyze the characteristics of the most-cited publications in the field of epilepsy epigenetics.

## Methods

The Web of Science (WoS) Core Collection database was used to list all papers and citations related to epilepsy epigenetics on January 19, 2023. The search terms used in the “Core Collection” section at Web of Science were as follows: TS = epilepsy epigenetics OR TS = seizure epigenetics OR TS = epilepsy epigenomics OR TS = seizure epigenomics OR TS = epilepsy DNA methylation OR TS = epilepsy histone modification OR TS = epilepsy chromatin remodeling OR TS = epilepsy microrna OR TS = epilepsy circular RNA OR TS = epilepsy LncRNA OR TS = epilepsy SiRNA OR TS = seizure DNA methylation OR TS = seizure histone modification OR TS = seizure chromatin remodeling OR TS = seizure microrna OR TS = seizure circular RNA OR TS = seizure LncRNA OR TS = seizure SiRNA.

The final interrogation was performed independently by two researchers. The search was limited to English language, articles, or reviews. Exclusion criteria were articles in languages other than English and those in which the main focus of the study was not directly relevant to epilepsy epigenetics.

The 100 articles with the most citations were further analyzed by publication year, citation density, authorship, journal, country, institution, manuscript type, theme, and clinical topics. Microsoft Excel 2019 software and VOSviewer (1.6.19) were used. The synonyms were merged, and thresholds of elements and the counting method were settled according to the operation manual of VOSviewer. In network visualization, the size of the nodes reflects the frequency of co-occurrence, and the size of the lines between nodes represents the strength between two nodes. The colors of the nodes indicate the different clusters. In overlay visualization, items are colored based on their average appearing time. Nodes in purple or blue appear earlier than those in yellow. The 2021 impact factor of each journal was identified using the Journal Citation Reports dataset.

A potential bias for this type of study is that older manuscripts accrue a higher number of citations over time. Therefore, the citation rate was calculated by dividing the number of citations by the number of years since publication. All of the papers from these journals were ranked according to their citation number, and articles accruing equal numbers of citations were ranked by citation rates.

## Result

The Web of Science search returned a total of 1231 manuscripts. Table S1 lists the 100 most-cited articles as ranked by citation number after the application of exclusion criteria. The median number of citations for a manuscript was 129, ranging from 739 to 75. The most-cited manuscript, with a total of 739 citations, was by Aid et al. published in the *Journal of Neuroscience Research* in 2007, which elaborated a new nomenclature for mouse and rat BDNF gene and its expression profiles [11].

**Fig. 2:**
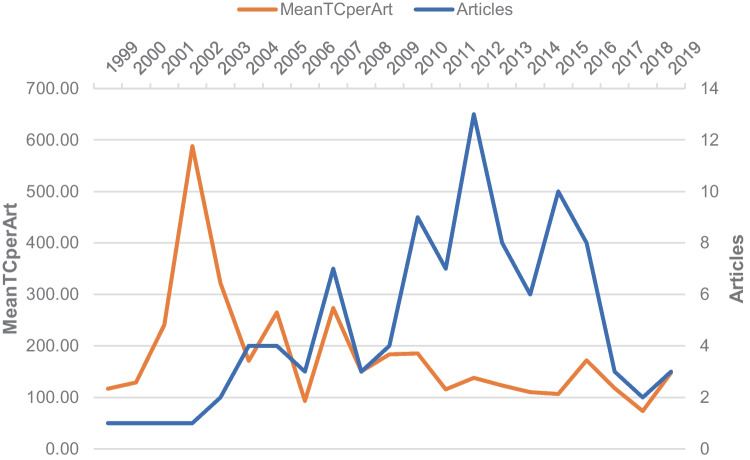
Line chart of top 100 most-cited articles according to the publishing time. MeanTCperART, mean total citation per article

The top 100 most-cited manuscripts were published across a range of decades, with numbers increasing and fluctuating from 1999 onwards to a peak between 2010 and 2016, as demonstrated in Fig. 2. The most historic paper was that by Fang P et al. which reported UBE3A mutations in 17 patients diagnosed of Angelman syndrome with normal DNA methylation analysis and their genealogy characteristics, published in the *Human Molecular Genetics* in 1999, and was cited 124 times [12]. The most recent manuscripts were by Juzwik, CA, published in 2019, which performed a systematic review of microRNA dysregulation in neurodegenerative diseases and related animal models [13].

A total of 632 scientists worldwide contributed to the T100 articles. The most prolific scientists with more than 3 T100 articles are displayed in Table 1. Professor Henshall DC ranked first with 11 T100 articles (2 as first author, 9 as corresponding author), and professor Jimenez-Mateos EM ranked second with 8 T100 articles (4 as first author), and they are both from the Royal College of Surgeons, Ireland.

**Table 1:** The most productive authors in T100 articles

Rank	Authors	Articles	TC	Average citations per article
1	Henshall DC	11	1358	123.5
2	Jimenez-Mateos EM	8	1000	125.0
3	Mckiernan RC	6	851	141.8
4	Sano T	6	831	138.5
5	Stallings RL	6	854	142.3
6	Blumcke I	4	449	112.3
7	Boison D	4	461	115.3
8	Engel T	4	671	167.8
9	Williams CA	4	657	164.3
10	Aronica E	3	627	209.0
11	Bray I	3	326	108.7
12	Gan N	3	304	101.3
13	Gorter JA	3	627	209.0
14	Hahnen E	3	337	112.3
15	Hauke J	3	337	112.3
16	Iyer A	3	627	209.0
17	Kobow K	3	338	112.7
18	Kong HM	3	304	101.3
19	Omran A	3	304	101.3
20	Peng J	3	304	101.3
21	Simon RP	3	326	108.7
22	Yin F	3	304	101.3

*TC* total citations

**Fig. 3:**
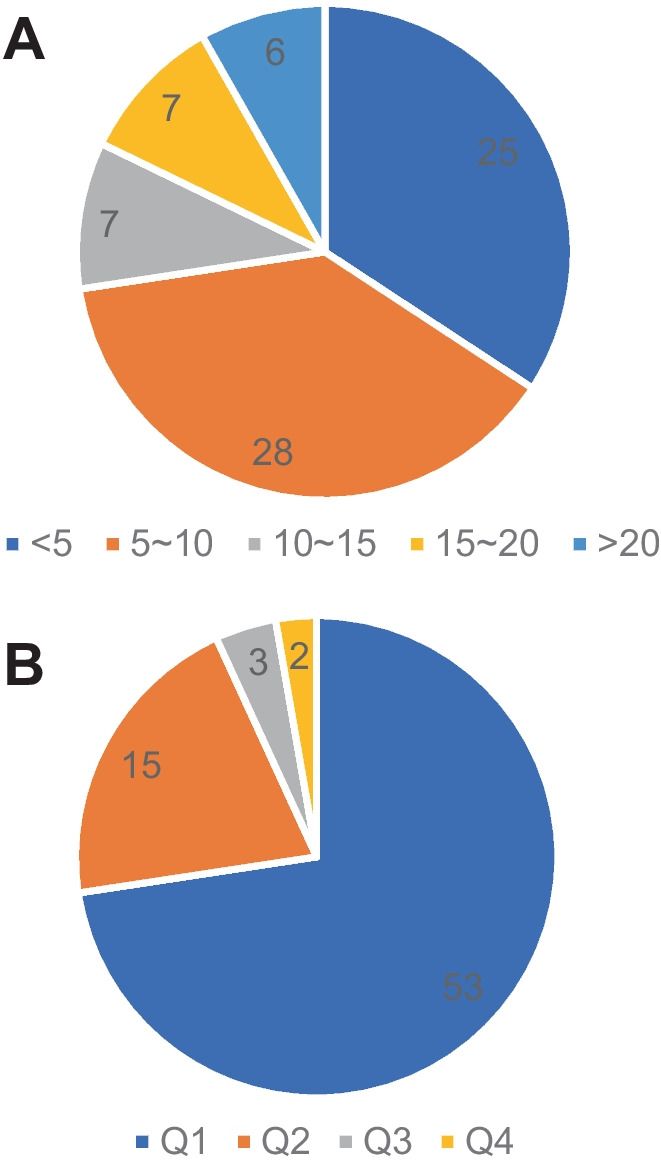
Pie chart demonstrating distribution of the top 100 most-cited articles according to the 2021 impact factor of the journal (**A**), and category quartile in which they are published (**B**)

The most relevant journals with more than 2 T100 manuscripts are given in Table 2. The greatest number was published in the *Human Molecular Genetics*, which had a 2021 impact factor of 5.121 (*n *= 4616 total citations), and *Neurobiology of Disease* (*n *= 4402 total citations, IF = 7.046), followed by *Cellular and Molecular Life Sciences* (*n *= 3462 total citations, IF = 9.234), *Journal of Medical Genetics* (*n *= 3701 total citations, IF = 5.945), and *Journal of Molecular Neuroscience* (*n *= 3276 total citations, IF = 2.866). The journal with the highest 2021 impact factor was *Nature Medicine* (IF = 87.244), which published 1 of the T100 manuscripts that primarily verified the neuroprotective and seizure-suppressive effects of silencing microRNA-134 in vivo [14]. The distribution of manuscripts according to the impact factor and JCR category quartile of the publishing journal is given in Fig. 3, which can be seen that 92% of manuscripts were published in journals with IF of > 3.0. Moreover, 93.1% of manuscripts were published in Q1 or Q2.

**Table 2:** Journal distribution of T100 articles in epilepsy epigenetics

Rank	Sources	Articles	IF	Total number of citations	Average citations per article	PY
1	*Human Molecular Genetics*	4	5.121	616	25.67	1999
2	*Neurobiology of Disease*	4	7.046	402	30.92	2010
3	*Cellular and Molecular Life Sciences*	3	9.234	462	28.88	2007
4	*Journal of Medical Genetics*	3	5.945	701	31.86	2001
5	*Journal of Molecular Neuroscience*	3	2.866	276	25.09	2012
6	*Acta Neuropathologica*	2	15.887	243	24.30	2013
7	*Cell Death and Disease*	2	9.696	156	14.18	2012
8	*Current Opinion in Neurology*	2	6.283	231	25.67	2014
9	*Developmental Dynamics*	2	2.842	241	16.07	2008
10	*Epilepsia*	2	6.740	193	16.08	2011
11	*European Journal of Neuroscience*	2	3.698	327	19.24	2006
12	*Frontiers in Cellular Neuroscience*	2	6.147	206	22.89	2014
13	*Journal of Biological Chemistry*	2	5.485	400	17.39	2000
14	*Journal of Neuroscience*	2	6.709	425	22.37	2004
15	*Lancet Neurology*	2	59.935	568	40.57	2009
16	*Neuron*	2	18.688	1179	56.14	2002
17	*Neuropharmacology*	2	5.273	164	20.50	2015
18	*Neuroscience*	2	3.708	347	34.70	2013
19	*PLOS One*	2	3.752	335	30.45	2012
20	*Scientific Reports*	2	4.997	176	22.00	2015

*PY* publication year

The most relevant country by the corresponding author of T100 articles is demonstrated in Table 3. The country with the highest number of manuscripts was the USA, followed by China. The USA dominated the area with 45 T100 articles and 7830 total citations. In aspect to multi-country cooperation, Ireland was outstanding with 11 manuscripts out of T100 and the MCP ratio was 0.727, which means they attach great importance to transnational cooperation. In total, 174 different institutions contributed to those articles; of those published, more than 7 are represented in Table 4. Most of those 9 institutions are located in the USA (*n *= 4), followed by China (*n *= 2) and the Netherlands (*n *= 2). However, the Royal College of Surgeons from Ireland leads the list with 19 T100 articles.

**Table 3:** Countries of origin for the T100 articles in epilepsy epigenetics

Country	Articles	SCP	MCP	MCP ratio	Total citations	Average article citations
USA	45	36	9	0.2	7830	174
China	12	8	4	0.333	1163	96.92
Ireland	11	3	8	0.727	1358	123.45
Germany	5	3	2	0.4	554	110.8
Netherlands	5	3	2	0.4	916	183.2
Canada	4	4	0	0	773	193.25
Italy	3	2	1	0.333	431	143.67
France	2	1	1	0.5	193	96.5
Israel	2	2	0	0	307	153.5
UK	2	1	1	0.5	475	237.5
Australia	1	1	0	0	140	140
Chile	1	0	1	1	82	82
Estonia	1	1	0	0	715	715
Japan	1	1	0	0	79	79
Luxembourg	1	1	0	0	240	240
Mexico	1	1	0	0	120	120
Singapore	1	0	1	1	157	157
Slovenia	1	1	0	0	201	201
Spain	1	1	0	0	415	415

*SCP* single country participation, *MCP* multiple country participation

**Table 4:** Top publishing institutions

Rank	Affiliation	Articles	Country
1	Royal Coll Surgeons Ireland	19	Ireland
2	Univ Florida	11	USA
3	Baylor Coll Med	10	USA
4	Cent S Univ	9	China
5	Capital Med Univ	8	China
6	Columbia Univ	7	USA
7	Univ Amsterdam	7	Netherlands
8	Univ Med CTR Utrecht	7	Netherlands
9	Univ Texas	7	USA

**Fig. 4:**
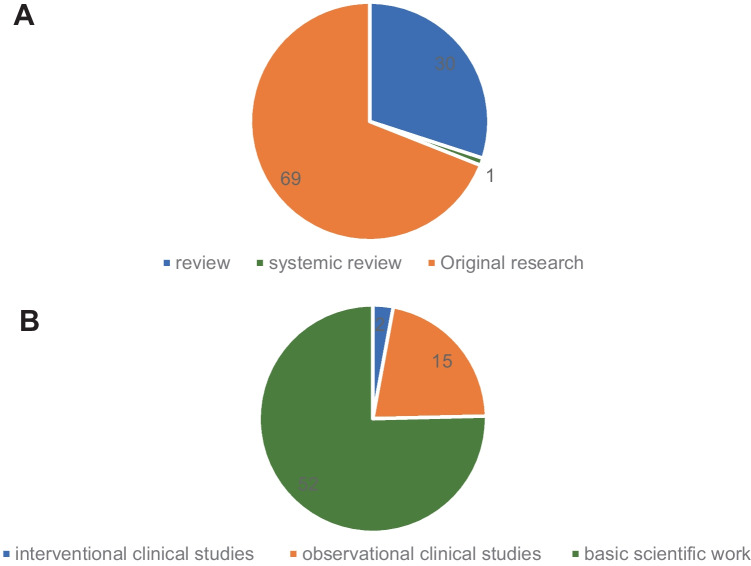
Pie chart demonstrating the study design of the top 100 articles (**A**) and the composition of original research articles (**B**)

**Fig. 5:**
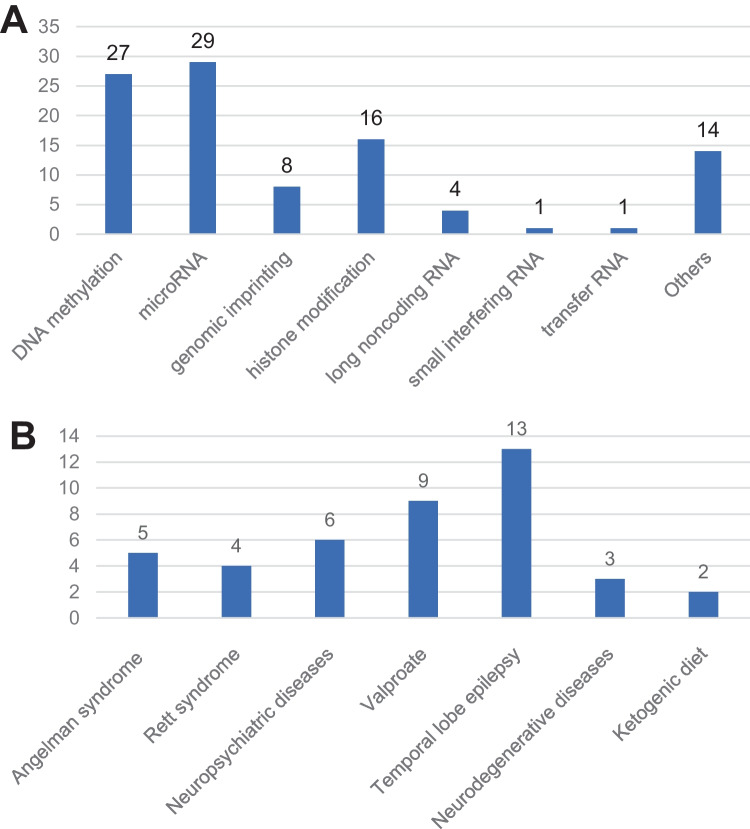
Bar graph demonstrating the theme (**A**) and clinical topic (**B**) of epilepsy epigenetics in the top 100 most-cited articles

The distribution of manuscripts according to study design is demonstrated in Fig. 4. Most were original articles (*n* = 69), of which 52 (75.4%) report findings of basic scientific work, 15 (21.7%) report observational clinical studies (i.e., clinical trials), and the remaining 2 (2.9%) report interventional clinical studies. The number of manuscripts relating to each theme within the topic of epilepsy epigenetics is given in Fig. 5A. The majority were focused on *microRNA* (*n* = 29), followed by *DNA methylation* (*n* = 27) and *histone modification* (*n* = 16), respectively. There were 14 articles that cannot be classified because they were cross-categories, or discussing the epigenetic function of *BET protein family* [15], *ADAR protein family* [16], and *short-chain fatty acids* [17]. Regardless of the clinical topic that was the focus, most of the T100 articles were related to *temporal lobe epilepsy* (*n *= 13), followed by *VPA* (*n *= 9), *neuropsychiatric diseases* (*n *= 6), and *Angelman syndrome* (*n *= 5), as shown by Fig. 5B.

**Fig. 6:**
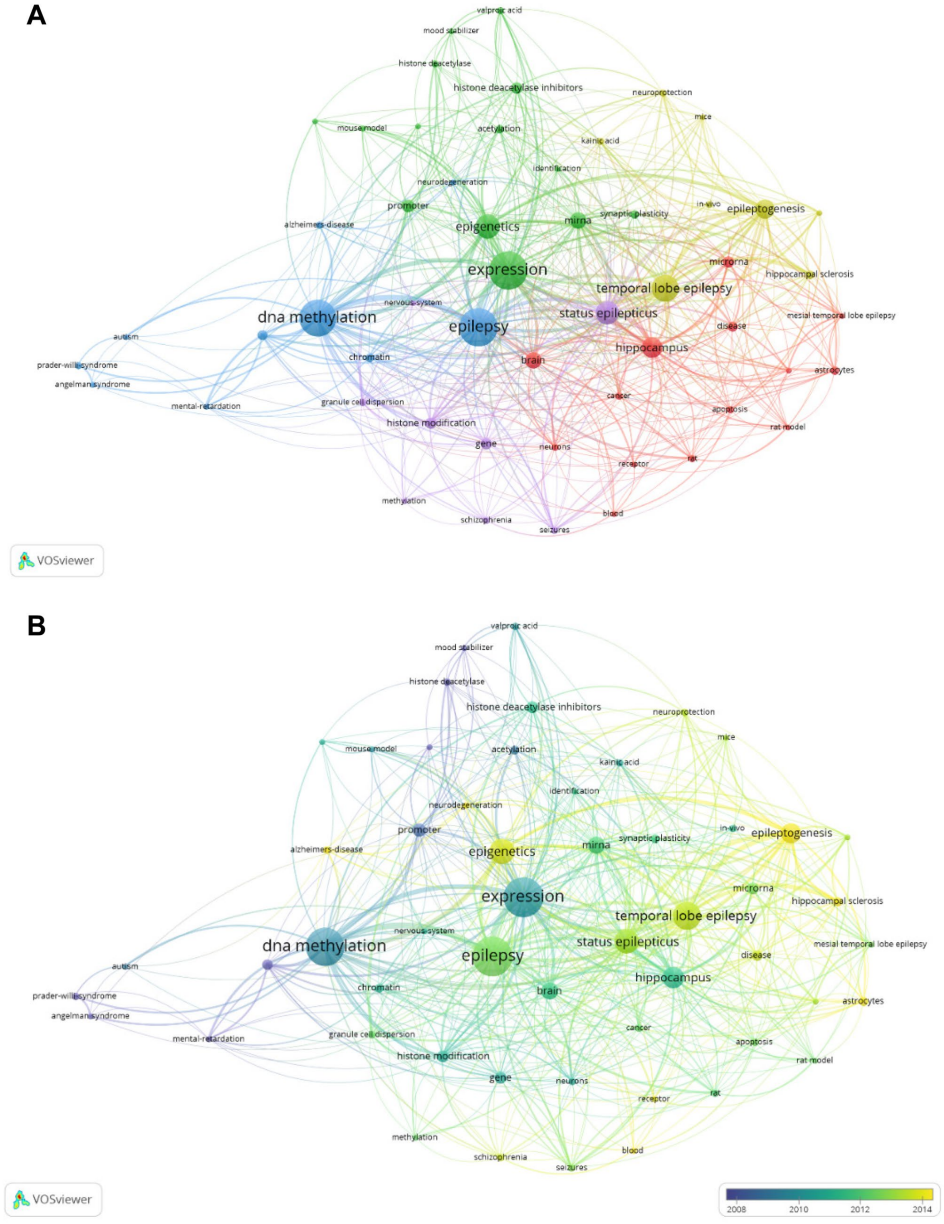
Network plot of influential keywords in epilepsy epigenetics research among the top‐100 cited articles of the WoSCC database. **A** Network visualization. **B** Overlay visualization of **A**

To determine influential keywords among the T100 articles on the WoSCC database, we performed a network analysis. The full counting method was adopted by VOSviewer. After merging synonyms, the co-occurrences of all keywords are calculated and 54 items with a minimum of 4 co-occurrences were selected to create a network visualization (Fig. 6A). Based on the results of co-occurrences, the top 100 most-cited articles in epilepsy epigenetics research mainly focused on *DNA methylation*, *miRNA*, *temporal lobe epilepsy*, *histone modifications*, and *hippocampus*. And they were identified as five clusters. The overlay visualization (Fig. 6B) showed that *astrocytes*, *epileptogenesis*, *hippocampal sclerosis*, and *Alzheimer disease* were the recent hotspots.

**Fig. 7:**
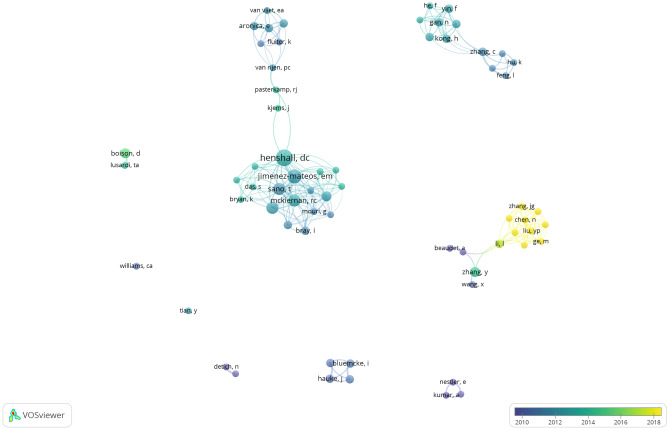
Overlay visualization map of top authors in epilepsy epigenetics research. Some names might be invisible because of names overlapping

We further performed a network analysis of co-authorship. The units of analysis were authors and a fractional counting method was adopted. Figure 7 demonstrates the overlay visualization of 66 authors with a minimum of 2 documents. The top researcher of epilepsy epigenetics could be roughly classified into 9 groups. Among them, the Royal College of Surgeons in Ireland had the largest research team consisting of Henshall DC, Jimenez-Mateos EM, McKiernan RC, and their fellows. They had close cooperation with Aronica E, Iyer A, and Gorter JA, who worked for the University of Amsterdam in the Netherlands. The research team focusing on epilepsy epigenetics recently was the Beijing Children’s Hospital, Capital Medical University in China, mainly comprised of Chen, N, Liu, YP, and Han, CL.

## Discussion

In this study, we evaluated the so far 100 most influential papers related to epilepsy epigenetics. A highly cited paper can be seen as a milestone of a given field. Thus, the results of the current study not only present historical scientific progress in epilepsy epigenetics but also display key trends and achievements in this field.

A concentration of publications between 2010 and 2016 could be seen. However, usually 1 or 2 years after publication, scientific papers start to be cited and about 10 years after the date of publication they reach a citation peak [18], which may represent a late consolidation of research in epigenetics of epilepsy.

The term “impact factor” represents the average number of citations of manuscripts pertaining to a journal within a given time period. As in our research, most of the T100 articles were included in journals with IF of ≥ 3.0 and high ranking in the category, which is an important indicator that key papers relating to epilepsy epigenetics are preferred by high-quality journals.

The USA is the dominant country in the research of epilepsy epigenetics, with the largest number of T100 articles, scientists, and institutions. This was probably because some of the world’s leading laboratories are located there and they fund a lot to scientific research. Nevertheless, the most‐cited author (Henshall DC) in the T100 list and the dominant research group are from Ireland. Together, China and Ireland accounted for 23% of the most‐cited papers, reflecting that these countries had made significant contributions to the research in this field.

The article *Epigenetic mechanisms in neurological diseases: genes, syndromes, and therapies* by Urdinguio RG et al. published in the journal *Lancet Neurology* in 2009 had the highest number of citations (ranked 5th) for a review. It comprehensively illustrated the contribution of DNA methylation and histone modifications to neurological diseases, such as Rett syndrome, Alzheimer disease, multiple sclerosis, and epilepsy. Moreover, it discussed the therapeutic potential of epigenetic drugs [19].

Only 2 interventional clinical studies were found in this research. The manuscript published by Kobow K et al. in 2009 indicated that epigenetic silencing by reelin promoter methylation was an epigenetic mechanism in the pathogenesis of TLE [20]. Other interventional clinical studies were by Braiteh et al. They conducted a phase I study of the combination of 5-azacytidine and valproic acid in patients with advanced malignancies. The therapeutic effect and safety dose were explored preliminarily [21].

According to the study design of the top 100 articles, most of the manuscripts were original research and based on basic science. No guideline was found in this list. The scarcity of clinical studies and guidelines reflected a long distance of this issue from the laboratory to the clinic. Only 1 systemic review ranked 27th within the T100 articles. This manuscript, published by Juzwik CA et al. in 2019, compiled the results from studies of microRNA expression in neurodegenerative diseases. Common pathways targeted by miRNAs were identified, and overlap pathological features across diseases were revealed. The understanding of commonalities and potential biomarkers in neurodegenerative disease pathogenesis was improved [13].

The network analysis of influential keywords among the T100 articles showed the interest of researchers relevant to epilepsy epigenetics research. A majority of articles (*n *= 29) were discussing *miRNA*. In 2010, Liu DZ et al. found that miRNA response profiles were different for the injured hippocampus and the controls, indicating the possible use of blood miRNAs as biomarkers for brain injury [22]. In the same year, Aronica E et al. reported prominent upregulation of miR-146a in rat hippocampus at 1 week after induction of status epilepticus and persisted in the chronic phase [23]. The subsequent studies revealed more than 20 miRNAs expressed differentially in the brain tissue of TLE rats or humans, including miR-132, miRNA-134, miR-23a/b, miRNA-146a, miR-34a, miR-184, miR-124, miR-21, and miRNA-155 [14, 24–30]. Identifying the exact pathway and target of miRNAs, finding a stable and safe approach to deliver large molecular miRNAs to the brain of patients, and conducting more randomized, blinded, intent-to-treat trials will be the future task and challenges [31].

*DNA methylation* was the second-hottest research theme in T100 articles (*n *= 27). It consists of the covalent addition of a methyl group at the 5-position of cytosines that are followed by guanines (CpG dinucleotides) [19]. Rett syndrome is caused by mutations in X-linked MECP2, encoding methyl-CpG-binding protein 2. The latter binds to methylated cytosines at CpG dinucleotides, as well as to unmethylated DNA, and affects chromatin condensation [32]. ATRX gene mutations cause diverse changes in the pattern of DNA methylation at heterochromatic loci, which result in X-linked alpha thalassaemia mental retardation syndrome, and seizures occur in about one-third of the cases [33]. DNA methylation had also been reported to wide participants in the therapeutic mechanism of choline, VPA, and the ketogenic diet [34–36].

TLE may develop as a result of brain trauma, infection, status epilepticus, or other brain insults, which is the most common syndrome in adults and prone to be pharmacoresistance. The epigenetics of TLE is the most popular research topic in T100 articles. Scattered observational evidence supports that epigenetic changes in TLE were associated with cell proliferation and immune and inflammatory responses [37]. The underlying epigenetic mechanisms include hypermethylation of promoters [38], over-expression of DNA methyltransferase [39], modulation of miRNA, and lncRNA activation [29, 40], suggesting novel therapeutic targets to treat. However, the exact function pathway and targets of those regulatory molecules should be identified before it can be widely used in interventional studies [31].

Angelman syndrome (AS) is a neurodevelopmental disorder characterized by severe mental disability, ataxia, seizure, and a happy, sociable disposition. It is caused by a variety of genetic abnormalities involving the chromosome 15q11-13 region, which is subject to genomic imprinting [41]. Lossie et al. analyzed 104 patients in 2001 and found the patients with deletion of 15q11–q13 had the most severe phenotype, while patients with paternal uniparental disomy and imprinting defect suffered relatively mild [42]. Besides, the concrete mechanisms underlying genomic imprinting were yet to be explored.

Our study has limitations. First, the keywords of epigenetics are fragmentary and scattered. The total number of search results of this research (*n* = 1231) was smaller than other citation analysis projects [7, 43, 44]; except for the fact that this issue was in the early stage and the lack of research achievements, some articles discussing epigenetics of epilepsy while they do not contain pertinent keywords may have been missed. Second, only articles with the top 100 total citations were selected. The historic articles may receive a higher number of citations because of the duration of availability to be cited, while the latest publication with a strong impact might be ignored. Furthermore, manuscripts may be cited repeatedly because of professional bias, self-citation, and institutional and language biases, which likely affected our results.

## Conclusion

We identified the most influential articles in epilepsy epigenetics. The research of epilepsy epigenetics was in its infancy but has received a lot of attention. The USA made the most contributions in terms of publication numbers, authors, and institutions. The landmark manuscripts were reviewed. The developmental history and current achievements of hot topics, including microRNA, DNA methylation, and temporal lobe epilepsy, were overviewed. The unsolved problems and future directions were carried out. This bibliometric analysis provides valuable insight into the research conducted in this field.

### Supplementary Information

Below is the link to the electronic supplementary material.

Supplementary file1 (DOCX 32 KB)

## Data Availability

The authors confirm that the data supporting the findings of this study are available within the article and its supplementary materials.
