# Supplementary material for: The 100 most-cited manuscripts in epilepsy epigenetics: a bibliometric analysis
Source: Childs Nerv Syst. 2023 Jun 20;39(11):3111–22. doi: 10.1007/s00381-023-06032-w (PMC10643235; doi:10.1007/s00381-023-06032-w)
Supplement: Supplementary file 1 — Supplementary file1 (DOCX 32 KB) [file 381_2023_6032_MOESM1_ESM.docx]

**Supplementary Material**

Table S1. The 100 most cited papers in epilepsy epigenetics.

| Rank | Authors | Article Title | Publication Year | Citation | Citation Rate |
| --- | --- | --- | --- | --- | --- |
| 1 | Aid, T | Mouse and rat BDNF gene structure and expression revisited | 2007 | 739 | 46.2 |
| 2 | Kumar, A | Chromatin remodeling is a key mechanism underlying cocaine-induced plasticity in striatum | 2005 | 623 | 34.6 |
| 3 | Shahbazian, MD | Mice with truncated MeCP2 recapitulate many Rett syndrome features and display hyperacetylation of histone H3 | 2002 | 600 | 28.6 |
| 4 | Liu, DZ | Brain and blood microRNA expression profiling of ischemic stroke, intracerebral hemorrhage, and kainate seizures | 2010 | 442 | 34.0 |
| 5 | Urdinguio, RG | Epigenetic mechanisms in neurological diseases: genes, syndromes, and therapies | 2009 | 427 | 30.5 |
| 6 | Clayton-Smith, J | Angelman syndrome: a review of the clinical and genetic aspects | 2003 | 391 | 19.6 |
| 7 | Tsankova, NM | Histone modifications at gene promoter regions in rat hippocampus after acute and chronic electroconvulsive seizures | 2004 | 359 | 18.9 |
| 8 | Jimenez-Mateos, EM | Silencing microRNA-134 produces neuroprotective and prolonged seizure-suppressive effects | 2012 | 358 | 32.5 |
| 9 | Tahiliani, M | The histone H3K4 demethylase SMCX links REST target genes to X-linked mental retardation | 2007 | 338 | 21.1 |
| 10 | Xie, ZY | Metabolic Regulation of Gene Expression by Histone Lysine beta-Hydroxybutyrylation | 2016 | 327 | 46.7 |
| 11 | Luedi, PP | Computational and experimental identification of novel human imprinted genes | 2007 | 293 | 18.3 |
| 12 | Detich, N | Valproate induces replication-independent active DNA demethylation | 2003 | 283 | 14.2 |
| 13 | Menard, C | PATHOGENESIS OF DEPRESSION: INSIGHTS FROM HUMAN AND RODENT STUDIES | 2016 | 268 | 38.3 |
| 14 | Aronica, E | Expression pattern of miR-146a, an inflammation-associated microRNA, in experimental and human temporal lobe epilepsy | 2010 | 263 | 20.2 |
| 15 | Iyer, A | MicroRNA-146a: A Key Regulator of Astrocyte-Mediated Inflammatory Response | 2012 | 259 | 23.5 |
| 16 | Mari, F | CDKL5 belongs to the same molecular pathway of MeCP2 and it is responsible for the early-onset seizure variant of Rett syndrome | 2005 | 257 | 14.3 |
| 17 | Nudelman, AS | Neuronal Activity Rapidly Induces Transcription of the CREB-Regulated MicroRNA-132, In Vivo | 2010 | 251 | 19.3 |
| 18 | Lossie, AC | Distinct phenotypes distinguish the molecular classes of Angelman syndrome | 2001 | 251 | 11.4 |
| 19 | Chateauvieux, S | Molecular and Therapeutic Potential and Toxicity of Valproic Acid | 2010 | 249 | 19.2 |
| 20 | Rosenberg, G | The mechanisms of action of valproate in neuropsychiatric disorders: can we see the forest for the trees? | 2007 | 234 | 14.6 |
| 21 | Piletic, K | MicroRNA epigenetic signatures in human disease | 2016 | 220 | 31.4 |
| 22 | Barrett, RM | Beyond transcription factors: The role of chromatin modifying enzymes in regulating transcription required for memory | 2008 | 207 | 13.8 |
| 23 | Milutinovic, S | Valproate induces widespread epigenetic reprogramming which involves demethylation of specific genes | 2007 | 203 | 12.7 |
| 24 | Tan, CL | MicroRNA-128 Governs Neuronal Excitability and Motor Behavior in Mice | 2013 | 201 | 20.1 |
| 25 | Williams, CA | Clinical and genetic aspects of Angelman syndrome | 2010 | 199 | 15.3 |
| 26 | Williams-Karnesky, RL | Epigenetic changes induced by adenosine augmentation therapy prevent epileptogenesis | 2013 | 182 | 18.2 |
| 27 | Juzwik, CA | microRNA dysregulation in neurodegenerative diseases: A systematic review | 2019 | 180 | 45.0 |
| 28 | Savva, YA | The ADAR protein family | 2012 | 175 | 15.9 |
| 29 | Jimenez-Mateos, EM | miRNA Expression Profile after Status Epilepticus and Hippocampal Neuroprotection by Targeting miR-132 | 2011 | 171 | 14.3 |
| 30 | Sun, WJ | Histone Acetylome-wide Association Study of Autism Spectrum Disorder | 2016 | 161 | 23.0 |
| 31 | Walton, E | Correspondence of DNA Methylation Between Blood and Brain Tissue and Its Application to Schizophrenia Research | 2016 | 160 | 22.9 |
| 32 | Kan, AA | Genome-wide microRNA profiling of human temporal lobe epilepsy identifies modulators of the immune response | 2012 | 159 | 14.5 |
| 33 | Braun, PR | Genome-wide DNA methylation comparison between live human brain and peripheral tissues within individuals | 2019 | 158 | 39.5 |
| 34 | Ansari, R | Hyperhomocysteinemia and Neurologic Disorders: a Review | 2014 | 158 | 17.6 |
| 35 | Hu, K | MicroRNA expression profile of the hippocampus in a rat model of temporal lobe epilepsy and miR-34a-targeted neuroprotection against hippocampal neurone cell apoptosis post-status epilepticus | 2012 | 158 | 14.4 |
| 36 | Henshall, DC | MicroRNAs in epilepsy: pathophysiology and clinical utility | 2016 | 157 | 22.4 |
| 37 | Korb, E | BET protein Brd4 activates transcription in neurons and BET inhibitor Jq1 blocks memory in mice | 2015 | 157 | 19.6 |
| 38 | Talkowski, ME | Assessment of 2q23.1 Microdeletion Syndrome Implicates MBD5 as a Single Causal Locus of Intellectual Disability, Epilepsy, and Autism Spectrum Disorder | 2011 | 156 | 13.0 |
| 39 | Lipovich, L | Activity-Dependent Human Brain Coding/Noncoding Gene Regulatory Networks | 2012 | 153 | 13.9 |
| 40 | Loke, YJ | The role of epigenetic change in autism spectrum disorders | 2015 | 144 | 18.0 |
| 41 | Chung, DW | A natural antisense transcript at the Huntington's disease repeat locus regulates HTT expression | 2011 | 144 | 12.0 |
| 42 | Kerkhof, M | Effect of valproic acid on seizure control and on survival in patients with glioblastoma multiforme | 2013 | 141 | 14.1 |
| 43 | Gao, BN | Functional properties of a new voltage-dependent calcium channel alpha(2)delta auxiliary subunit gene (CACNA2D2) | 2000 | 141 | 6.1 |
| 44 | Boison, D | New insights into the mechanisms of the ketogenic diet | 2017 | 140 | 23.3 |
| 45 | Gorter, JA | Hippocampal subregion-specific microRNA expression during epileptogenesis in experimental temporal lobe epilepsy | 2014 | 139 | 15.4 |
| 46 | Reid, G | Multiple mechanisms induce transcriptional silencing of a subset of genes, including oestrogen receptor alpha, in response to deacetylase inhibition by valproic acid and trichostatin A | 2005 | 136 | 7.6 |
| 47 | Kobow, K | Deep sequencing reveals increased DNA methylation in chronic rat epilepsy | 2013 | 135 | 13.5 |
| 48 | MacDonald, JL | Histone deacetylases 1 and 2 are expressed at distinct stages of neuro-glial development | 2008 | 135 | 9.0 |
| 49 | Kobow, K | Increased Reelin Promoter Methylation Is Associated With Granule Cell Dispersion in Human Temporal Lobe Epilepsy | 2009 | 129 | 9.2 |
| 50 | Braiteh, F | Phase I Study of Epigenetic Modulation with 5-Azacytidine and Valproic Acid in Patients with Advanced Cancers | 2008 | 129 | 8.6 |
| 51 | Kolesnikova, OA | Nuclear DNA-encoded tRNAs targeted into mitochondria can rescue a mitochondrial DNA mutation associated with the MERRF syndrome in cultured human cells | 2004 | 129 | 6.8 |
| 52 | Myers, CT | Advancing epilepsy genetics in the genomic era | 2015 | 128 | 16.0 |
| 53 | Nelson-DeGrave, VL | Valproate potentiates androgen biosynthesis in human ovarian theca cells | 2004 | 128 | 6.7 |
| 54 | Landgrave-Gomez, J | Epigenetic mechanisms in neurological and neurodegenerative diseases | 2015 | 125 | 15.6 |
| 55 | Fang, P | The spectrum of mutations in UBE3A causing Angelman syndrome | 1999 | 124 | 5.2 |
| 56 | Omran, A | Interleukin-1 beta and microRNA-146a in an immature rat model and children with mesial temporal lobe epilepsy | 2012 | 122 | 11.1 |
| 57 | Zaits, MN | Identification of differentially expressed microRNAs across the developing human brain | 2014 | 121 | 13.4 |
| 58 | Hsieh, J | Epigenetics, hippocampal neurogenesis, and neuropsychiatric disorders: Unraveling the genome to understand the mind | 2010 | 118 | 9.1 |
| 59 | Huse, JT | Polymorphous low-grade neuroepithelial tumor of the young (PLNTY): an epileptogenic neoplasm with oligodendroglioma-like components, aberrant CD34 expression, and genetic alterations involving the MAP kinase pathway | 2017 | 116 | 19.3 |
| 60 | Shang, EY | Double Bromodomain-Containing Gene Brd2 Is Essential for Embryonic Development in Mouse | 2009 | 116 | 8.3 |
| 61 | Jiang, YH | A mixed epigenetic/genetic model for oligogenic inheritance of autism with a limited role for UBE3A | 2004 | 116 | 6.1 |
| 62 | Peng, J | Expression Patterns of miR-124, miR-134, miR-132, and miR-21 in an Immature Rat Model and Children with Mesial Temporal Lobe Epilepsy | 2013 | 115 | 11.5 |
| 63 | Romoli, M | Valproic Acid and Epilepsy: From Molecular Mechanisms to Clinical Evidences | 2019 | 112 | 28.0 |
| 64 | Song, YJ | Temporal lobe epilepsy induces differential expression of hippocampal miRNAs including let-7e and miR-23a/b | 2011 | 112 | 9.3 |
| 65 | Blusztajn, JK | Neuroprotective Actions of Dietary Choline | 2017 | 111 | 18.5 |
| 66 | Miller-Delaney, SFC | Differential DNA methylation profiles of coding and non-coding genes define hippocampal sclerosis in human temporal lobe epilepsy | 2015 | 109 | 13.6 |
| 67 | Feng, WH | Valproic acid enhances the efficacy of chemotherapy in EBV-positive tumors by increasing lytic viral gene expression | 2006 | 106 | 6.2 |
| 68 | Jordan, C | Cerebellar gene expression profiles of mouse models for Rett syndrome reveal novel MeCP2 targets | 2007 | 105 | 6.6 |
| 69 | Luo, Q | Long noncoding RNAs and Alzheimer's disease | 2016 | 104 | 14.9 |
| 70 | Wang, J | Genome-wide circulating microRNA expression profiling indicates biomarkers for epilepsy | 2015 | 103 | 12.9 |
| 71 | Gibbons, R | Alpha thalassaemia-mental retardation, X linked | 2006 | 103 | 6.1 |
| 72 | Lalande, M | Molecular epigenetics of Angelman syndrome | 2007 | 101 | 6.3 |
| 73 | Henshall, DC | MicroRNA and epilepsy: profiling, functions and potential clinical applications | 2014 | 100 | 11.1 |
| 74 | McKiernan, RC | Reduced Mature MicroRNA Levels in Association with Dicer Loss in Human Temporal Lobe Epilepsy with Hippocampal Sclerosis | 2012 | 100 | 9.1 |
| 75 | DeCastro, M | Short chain fatty acids regulate tyrosine hydroxylase gene expression through a cAMP-dependent signaling pathway | 2005 | 100 | 5.6 |
| 76 | Hu, K | Expression profile of microRNAs in rat hippocampus following lithium-pilocarpine-induced status epilepticus | 2011 | 98 | 8.2 |
| 77 | Ashhab, MU | Expressions of Tumor Necrosis Factor Alpha and MicroRNA-155 in Immature Rat Model of Status Epilepticus and Children with Mesial Temporal Lobe Epilepsy | 2013 | 97 | 9.7 |
| 78 | Jimenez-Mateos, EM | EPILEPSY AND MICRORNA | 2013 | 96 | 9.6 |
| 79 | Jiang, Y | miR-210 mediates vagus nerve stimulation-induced antioxidant stress and anti-apoptosis reactions following cerebral ischemia/reperfusion injury in rats | 2015 | 95 | 11.9 |
| 80 | Sano, T | MicroRNA-34a upregulation during seizure-induced neuronal death | 2012 | 91 | 8.3 |
| 81 | Banerjee, S | Genetic aspects of autism spectrum disorders: insights from animal models | 2014 | 90 | 10.0 |
| 82 | Hogart, A | Chromosome 15q11-13 duplication syndrome brain reveals epigenetic alterations in gene expression not predicted from copy number | 2009 | 90 | 6.4 |
| 83 | Sng, JCG | Histone modifications in kainate-induced status epilepticus | 2006 | 89 | 5.2 |
| 84 | Kobow, K | The methylation hypothesis: Do epigenetic chromatin modifications play a role in epileptogenesis? | 2011 | 88 | 7.3 |
| 85 | Sailaja, BS | Stress-induced epigenetic transcriptional memory of acetylcholinesterase by HDAC4 | 2012 | 87 | 7.9 |
| 86 | Boison, D | Adenosinergic signaling in epilepsy | 2016 | 86 | 12.3 |
| 87 | Aksoy-Aksel, A | MicroRNAs and synaptic plasticity-a mutual relationship | 2014 | 86 | 9.6 |
| 88 | Miller-Delaney, SFC | Differential DNA Methylation Patterns Define Status Epilepticus and Epileptic Tolerance | 2012 | 85 | 7.7 |
| 89 | Zhu, Q | Increased Expression of DNA methyltransferase 1 and 3a in Human Temporal Lobe Epilepsy | 2012 | 85 | 7.7 |
| 90 | Behrens, MI | Clinical Spectrum of Kufor-Rakeb Syndrome in the Chilean Kindred with ATP13A2 Mutations | 2010 | 85 | 6.5 |
| 91 | Han, CL | LncRNA H19 contributes to hippocampal glial cell activation via JAK/STAT signaling in a rat model of temporal lobe epilepsy | 2018 | 83 | 16.6 |
| 92 | Lusardi, TA | Ketogenic diet prevents epileptogenesis and disease progression in adult mice and rats | 2015 | 83 | 10.4 |
| 93 | Dagli, A | Molecular and Clinical Aspects of Angelman Syndrome | 2011 | 83 | 6.9 |
| 94 | Jimenez-Mateos, EM | Antagomirs targeting microRNA-134 increase hippocampal pyramidal neuron spine volume in vivo and protect against pilocarpine-induced status epilepticus | 2015 | 82 | 10.3 |
| 95 | Qureshi, IA | Epigenetic mechanisms underlying human epileptic disorders and the process of epileptogenesis | 2010 | 82 | 6.3 |
| 96 | Carouge, D | CDKL5 is a brain MeCP2 target gene regulated by DNA methylation | 2010 | 82 | 6.3 |
| 97 | McKiernan, RC | Expression profiling the microRNA response to epileptic preconditioning identifies miR-184 as a modulator of seizure-induced neuronal death | 2012 | 80 | 7.3 |
| 98 | Hwang, JY | Epigenetic Mechanisms in Stroke and Epilepsy | 2013 | 79 | 7.9 |
| 99 | Roncon, P | MicroRNA profiles in hippocampal granule cells and plasma of rats with pilocarpine-induced epilepsy - comparison with human epileptic samples | 2015 | 77 | 9.6 |
| 100 | Han, CL | Long non-coding RNA H19 contributes to apoptosis of hippocampal neurons by inhibiting let-7b in a rat model of temporal lobe epilepsy | 2018 | 75 | 15.0 |
